# Online health information seeking, low atrial fibrillation–related quality of life, and high perceived efficacy in patient-physician interactions in older adults with atrial fibrillation

**DOI:** 10.1016/j.cvdhj.2022.03.001

**Published:** 2022-03-11

**Authors:** Jordy Mehawej, Ajay Mishra, Jane S. Saczynski, Molly E. Waring, Darleen Lessard, Hawa O. Abu, Vincent La, Mayra Tisminetzky, Khanh-Van Tran, Essa Hariri, Andreas Filippaios, Tenes Paul, Apurv Soni, Weijia Wang, Eric Y. Ding, Benita A. Bamgbade, Joanne Mathew, Catarina Kiefe, Robert J. Goldberg, David D. McManus

**Affiliations:** ∗ Division of Cardiovascular Medicine, Department of Medicine, University of Massachusetts Medical School, Worcester, Massachusetts; † Department of Medicine, Saint Vincent Hospital, Worcester, Massachusetts; ‡ Department of Pharmacy and Health Systems Sciences, School of Pharmacy, Northeastern University, Boston, Massachusetts; § Department of Allied Health Sciences, University of Connecticut, Storrs, Connecticut; ‖ Department of Population and Quantitative Health Sciences, University of Massachusetts Medical School, Worcester, Massachusetts; ¶ Division of Geriatrics, Department of Medicine, University of Massachusetts Medical School, Worcester, Massachusetts; # Department of Medicine, Cleveland Clinic, Cleveland, Ohio

**Keywords:** Atrial fibrillation, Online health information seeking, Quality of life, Patient-physician interactions, Older adults

## Abstract

**Background:**

Little is known about online health information–seeking behavior among older adults with atrial fibrillation (AF) and its association with self-reported outcomes.

**Objective:**

To examine patient characteristics associated with online health information seeking and the association between information seeking and low AF-related quality of life and high perceived efficacy in patient-physician interaction.

**Methods:**

We used data from the SAGE-AF (Systematic Assessment of Geriatric Elements in AF) study, which includes older participants aged ≥65 years with AF and a CHA_2_DS_2_-VASc risk score ≥2. To assess online health information seeking, participants who reported using the Internet were asked at baseline if they used the Internet to search for advice or information about their health in the past 4 weeks (not at all vs at least once). Atrial Fibrillation Effect on Quality of Life and Perceived Efficacy in Patient-Physician Interactions questionnaires were used to examine AF-related quality of life (QOL) and patient-reported confidence in physicians. Logistic regression models were used to examine demographic and clinical factors associated with online health information seeking and associations between information seeking and low AF-related QOL (AFEQT <80) and high perceived efficacy for patient-physician interactions (PEPPI ≥45).

**Results:**

A total of 874 online participants (mean age 74.5 years, 51% male, 91% non-Hispanic White) were studied. Approximately 60% of participants sought health information online. Participants aged 74 years or older and those on anticoagulation were less likely, while those with a college degree were more likely, to seek online health information after adjusting for potential confounders. Participants who sought health information online, compared to those who did not, were significantly more likely to have a low AF-related QOL, but less likely to self-report confidence in patient-physician interaction (aOR = 1.56, 95% CI: 1.15–2.13; aOR = 0.68, 95% CI: 0.49–0.93, respectively).

**Conclusion:**

Clinicians should consider barriers to patient-physician interaction in older adults who seek health information online, encourage shared decision-making, and provide patients with a list of online resources for AF in addition to disease education plans to help patients manage their health.

**Key Findings**

• A considerable population of patients with AF sought health information online.
• Older participants and those on anticoagulation were less likely, while those with a college degree were more likely, to seek online health information.
• Participants who sought health information online were more likely to have a low AF-related quality of life, but less likely to self-report confidence in patient-physicians interaction
• Clinicians should consider barriers to patient-physician interaction and provide patients with a list of online resources for AF to help patients manage their health.

## Introduction

Atrial fibrillation (AF) is the most prevalent clinically significant cardiac arrhythmia, affecting nearly 6 million Americans and many more individuals worldwide.^1^, ^2^, ^3^ Patients with AF are at increased risk of morbidity and mortality, including the development of an ischemic stroke, sudden cardiac death, and heart failure.^4^ While effective treatments are available for patients with AF, anticoagulant regimens and lifestyle approaches to disease management can be challenging, and therefore many patients have questions about managing their health.^5^ With the increase in the availability of smartphones, computers, and tablets, technology adoption among older adults in the United States has increased markedly over the past 2 decades.^6^ A growing number of patients with AF now use the Internet as a key resource to search for medical information including AF symptoms, complications, and treatment.^7^, ^8^, ^9^

Patients often search online for health information in order to prepare for their physician’s consultation, validate it, or challenge its outcome.^10^ Although several prior studies have examined the reasons why patients seek online health information, no previous study has examined the quality of life (QOL) and confidence in physician interaction among older patients with AF who sought health information online as compared with those who do not report seeking such information. Understanding these associations can help clinicians develop tailored interventions to improve patient-physician interactions, encourage shared decision-making, and emphasize the importance of AF disease education plans.

Using data from a multicenter prospective cohort study, the SAGE (Systematic Assessment of Geriatric Elements)-AF, we examined patient characteristics associated with online health information seeking among online patients and the association between online health information seeking and low AF-related QOL and high self-efficacy for patient-physician interaction.

## Methods

### Study population

Details of the SAGE-AF study have been previously described.^11^^,^^12^ In brief, between 2016 and 2018, the SAGE-AF study enrolled participants from multiple clinical sites in Massachusetts and central Georgia. Eligibility criteria to be included in the SAGE-AF study consisted of the following: (1) aged ≥65 years; (2) having a CHA_2_DS_2_VASC risk score ≥ 2^13^; (3) having a scheduled ambulatory care visit at one of the study practices; and (4) electrocardiographic evidence of AF. Participants were not included if they had an absolute contraindication to oral anticoagulants (OAC), were non–English speakers, or were unable to provide informed consent. The Institutional Review Boards at each study site approved this study.

At study enrollment, participants completed an interviewer-administered questionnaire and trained research staff reviewed and collected data through the abstraction of electronic medical records. The present cross-sectional study included data from participants’ baseline assessment. Sociodemographic data included age, sex, level of education, race, and marital status. Participants’ clinical characteristics included (1) type of AF; (2) electrocardiography (ECG) findings at baseline; (3) time since AF diagnosis; (4) AF treatment approach (ie, rhythm or rate control); (5) hemoglobin values; (6) anticoagulation use (OAC); and (7) medical history. Participants’ bleeding and stroke risk scores were calculated using the HAS-BLED and CHA_2_DS_2_-VASc scoring systems. In addition, participants completed a comprehensive geriatric assessment. The Generalized Anxiety Disorder-7 Scale,^14^ the Patient Health Questionnaire-9,^15^ and the Montreal Cognitive Assessment^16^ were used to assess symptoms of anxiety, symptoms of depression, and cognitive performance, respectively. Frailty was assessed using the Cardiovascular Health Survey frailty scale,^17^ and social isolation was assessed by the Social Support Scale and Social Network Scale.^18^

### Assessment of online health information seeking

To assess online health information seeking, online participants were asked at baseline, “If you used the Internet in the past 4 weeks, how often have you used the Internet to look for advice or information about your health?” The responses were “More than once a day,” “Once a day,” “More than once a week but not every day,” “Once a week,” Less than once a week,” and “Not at all in the past 4 weeks.”^19^ Participants’ responses to online health information seeking were dichotomized as never vs ever during the past 4 weeks, owing to the skewed distribution of responses.

### Outcome measures (assessment of AFEQT and PEPPI questionnaires)

We used the Atrial Fibrillation Effect on Quality of Life (AFEQT) questionnaire, a 20-item questionnaire, to examine participants’ AF-related QOL at baseline. This validated questionnaire assesses participants’ perceived degree to which AF has affected their QOL in the past month.^20^ Scores were calculated by adding the responses to subscales including daily activities, treatment concerns, and symptoms experienced. Scores range between 0 and 100, with lower scores indicating lower AF-related QOL. Participants with a score <80 were classified as having lower vs higher (≥80) reported QOL.^20^

We used the Perceived Efficacy in Patient-Physician Interactions (PEPPI), a 10-item validated questionnaire, to measure self-efficacy in patient-physician interactions.^21^ Scores range between 5 and 50.^21^ Participants with a score ≥45 were classified as high perceived efficacy in patient-provider interactions, which corresponds to average responses of very or extremely confident.

### Statistical analysis

We compared the characteristics of participants who sought health information online to those who never did using *t* tests for continuous variables and the χ^2^ test for categorical variables.

A logistic regression analysis was used to examine participants’ sociodemographic and clinical characteristics associated with online health information seeking. To understand the impact of different patient characteristics with regard to online health information seeking, a model-building approach was used in the regression analysis. We adjusted for groups of variables based on their statistical significance in their independent association with online health information seeking. In model 1, we adjusted for sociodemographic variables (age, sex, and education). In model 2, we adjusted for model 1 variables and clinical and geriatric factors (medical history of peripheral vascular disease, cognitive impairment, Charlson comorbidity index) and AF-related variables (ECG at baseline, anticoagulation use, and CHA_2_DS_2_-VASc risk score).

Crude and multivariable adjusted logistic regression models were used to examine the association between online health information seeking and the study outcomes of low AF-related QOL and high perceived efficacy in patient-physician interactions. Model building was also used. We adjusted for groups of variables based on their statistical as well as their clinical significance. In model 1, we adjusted for several sociodemographic variables, including age, sex, race, and level of education. In the more comprehensive regression model 2, we added clinically relevant variables and several potential confounders, including cognitive impairment, medical history of peripheral vascular disease, ECG at baseline, and anticoagulation use.

We performed all the statistical analyses using SAS 9.4 (SAS Institute Inc., Cary, NC).

## Results

Of the 1244 older adults with AF enrolled in SAGE-AF, 70% (n = 874) reported using the Internet in the past 4 weeks. The study population consisted of these 874 older adults with AF. The mean age of study participants was 74.5 years, 47% were women, and approximately 50% had a college degree or higher. A total of 60% of participants had paroxysmal AF and nearly one-third of participants were cognitively impaired. Half of participants received care from an electrophysiologist.

### Characteristics of participants who seek health information online

Overall, 60% (n = 519) of online patients sought health information online in the past 4 weeks. Patients who sought health information online in the past 4 weeks were on average slightly younger, with lower average Charlson Comorbidity index scores; were more likely to have a college degree or higher education; and were less likely to be cognitively impaired than online adults who did not seek health information online (Table 1).

**Table 1:** Participants’ baseline characteristics by online health information seeking among older adults with atrial fibrillation

Characteristics Sociodemographics	Online health information seeking		
	Never (n = 355)	Ever (n = 519)	*P* value
Age (y), mean (SD)	75.5 (6.9)	73.4 (6.2)	<.001∗
Age group			
65–74	177 (49.9)	334 (64.4)	<.001∗
75–84	136 (38.3)	147 (28.3)	
85+	42 (11.8)	38 (7.3)	
Women	175 (49.3)	240 (46.2)	.37
Non-Hispanic White	323 (91.0)	471 (90.8)	.91
Married	213 (60.0)	325 (62.9)	.39
College graduate or more	147 (41.5)	314 (60.9)	<.001∗
Clinical			
Type of AF			
Paroxysmal	204 (57.5)	325 (62.6)	.32
Persistent	97 (27.3)	124 (23.9)	
Permanent	22 (6.2)	22 (4.2)	
Time (y) since AF diagnosis, mean (SD)	5.4 (4.0)	5.3 (4.5)	.43
ECG at baseline			
AF	102 (31.0)	129 (26.4)	<.01∗
NSR	123 (37.5)	200 (41.0)	
AF treatment approach			
Rhythm control	206 (58.0)	322 (62.0)	.23
On anticoagulation	311 (87.6)	425 (81.9)	.02∗
Medical history			
Anemia	115 (32.4)	139 (26.8)	.07
Asthma/COPD	92 (26.0)	116 (22.4)	.23
Diabetes	78 (22.0)	128 (24.7)	.36
Heart failure	124 (34.9)	157 (30.3)	.15
Hypertension	320 (90.1)	452 (87.1)	.16
Major bleeding	59 (16.6)	86 (16.6)	.99
Myocardial infarction	71 (20.0)	87 (16.8)	.22
Peripheral vascular disease	57 (16.1)	56 (10.8)	.02∗
Renal disease	94 (26.5)	115 (22.2)	.14
Stroke/TIA	36 (10.1)	37 (7.1)	.12
Hemoglobin (g/dL)	13.1 (1.7)	13.4 (1.8)	.19
Charlson comorbidity index, mean (SD)	6.0 (2.6)	5.5 (2.3)	<.01∗
Risk scores,† mean (SD)			
CHA_2_DS_2_-VASc	4.4 (1.5)	4.0 (1.6)	<.01∗
HAS-BLED	3.2 (1.1)	3.1 (1.0)	.16
Geriatric			
Anxiety (GAD7)	72 (20.3)	122 (23.5)	.26
Depression (PHQ9)	82 (23.1)	148 (28.5)	.07
Cognitive impairment (MOCA ≤23)	131 (36.9)	135 (26.0)	<.001∗
Social isolation	34 (9.6)	64 (12.3)	.20
Frailty	33 (9.3)	55 (10.6)	.08
Health behaviors			
Current smoker	5 (1.4)	14 (2.7)	.22
Provider type			
Internist	9 (2.5)	15 (2.9)	.94
Cardiologist	171 (48.2)	246 (47.4)	
Electrophysiologist	175 (49.3)	258 (49.7)	

Results are n (%) unless specified. Statistically significant *P* values are indicated with an asterisk.

AF = atrial fibrillation; COPD = chronic obstructive pulmonary disease; ECG = electrocardiography; GAD7 = Generalized Anxiety Disorder; MOCA = Montreal Cognitive Assessment; NSR = normal sinus rhythm; PHQ9 = Patient Health Questionnaire 9; TIA = transient ischemic attack.

† CHA_2_DS_2_-VASc assesses stroke risk; HAS-BLED assesses bleeding risk.

### Factors associated with online health information seeking

Participants ≥75 years old, as compared with those aged 65–74 years, and participants on anticoagulation, as compared to those who were not, were significantly less likely to seek health information online, after adjusting for a number of potentially confounding variables (Table 2; adjusted odds ratio [aOR] = 0.55, 95% CI: 0.32–0.96; aOR = 0.62, 95% CI: 0.40–0.97, respectively). Participants with a college degree or higher were more than twice as likely to seek health information online as compared with participants with less education (Table 2; aOR = 2.19, 95% CI: 1.61–2.96).

**Table 2:** Participant characteristics associated with online health information seeking among older adults with atrial fibrillation

Sociodemographics	N (%)	Crude OR (95% CI)	Online health information seeking∗	
			Model 1 Adjusted OR (95% CI)	Model 2 Adjusted OR (95% CI)
Age (years)				
65–74	334 (64.4)	Ref.	Ref.	Ref.
75–84	147 (28.3)	0.54 (0.39–0.73)†	0.59 (0.44–0.80)†	0.61 (0.43–0.87)†
85+	38 (7.3)	0.50 (0.31–0.82)†	0.45 (0.28–0.73)†	0.55 (0.32–0.96)†
Men (vs women)	279 (53.7)	1.14 (0.86–1.50)	0.96 (0.72–1.27)	1.07 (0.78–1.46)
Non-Hispanic White (vs other races/ethnicities)	471 (90.8)	0.83 (0.50–1.39)	0.96 (0.59–1.56)	0.80 (0.46–1.38)†
College graduate or more (vs less education)	314 (60.9)	2.30 (1.73–3.06)†	2.20 (1.65–2.92)†	2.19 (1.61–2.96)†
Clinical				
Peripheral vascular disease (vs no)	56 (10.8)	0.62 (0.41–0.93)†		0.63 (0.39–1.00)
CHA_2_DS_2_-VASc‡	4.0 (1.6)	0.87 (0.79–0.95)†		1.08 (0.94–1.24)
Charlson comorbidity index	5.46 (2.3)	0.91 (0.86–0.97)†		0.98 (0.91–1.06)
Geriatric				
Cognitive impairment (vs no)	135 (26.0)	0.62 (0.46–0.84)†		0.72 (0.52–1.01)
AF				
ECG at baseline				
NSR	200 (41.0)	Ref.		Ref.
AF	129 (26.4)	0.78 (0.55–1.10)		0.93 (0.65–1.35)
On anticoagulation (vs not)	425 (81.9)	0.59 (0.39–0.89)†		0.62 (0.40–0.97)†

AF = atrial fibrillation; ECG = electrocardiography; NSR = normal sinus rhythm; TIA = transient ischemic attack.

∗ Model 1: adjusting for sociodemographic factors; Model 2: M1 + clinical and geriatric factors + AF-related factors that differed significantly in Table 1.

† Statistically significant *P* values.

‡ CHA2DS2-VASc assesses stroke risk.

### Online health information seeking and the primary study outcomes

Participants who sought health information online, compared with those who did not, were significantly more likely to have a lower AF-related quality of life, after adjusting for several potentially confounding factors (Table 3; 44% vs 37%, aOR = 1.56; 95% CI: 1.15–2.13). Participants who sought health information online, compared with those who did not, were 32% less likely to report high efficacy in patient-physician interaction, after adjusting for several sociodemographic and clinical confounders (Table 3; 64% vs 72%, aOR = 0.68; 95% CI: 0.49–0.93).

**Table 3:** Association between online health information seeking and low atrial fibrillation–related quality of life and high perceived efficacy in patient-physician interactions among older adults with atrial fibrillation

	No online health information seeking, n (%)	Online health information seeking, n (%)	Unadjusted OR (95% CI)	Model 1 adjusted OR (95% CI)∗	Model 2 adjusted OR (95% CI)∗
Low AF-related QOL	131 (36.9)	228 (43.9)	1.34 (1.02–1.77)†	1.42 (1.06–1.90)†	1.56 (1.15–2.13)†
High PEPPI	250 (71.6)	325 (64.0)	0.70 (0.52–0.95)†	0.69 (0.51–0.93)†	0.68 (0.49–0.93)†

AF = atrial fibrillation; PEPPI = perceived efficacy for patient-physician interactions; QOL = quality of life.

∗ Model 1: adjusting for sociodemographic factors; Model 2: M1 + clinical and geriatric factors + AF-related factors that differed significantly in Table 1.

† Statistically significant *P* values.

## Discussion

In this multicenter cohort of older adults with AF, we found that 60% of online participants sought health information online, similar to rates among older adults hospitalized with acute coronary syndrome and older postmenopausal women.^22^^,^^23^ Older participants (≥74 years) and those on anticoagulation therapy were less likely to seek health information online, while participants with at least a college degree were more likely to do so. Online adults with AF who sought health information online were more likely to have a low AF-related QOL and less likely to report high perceived efficacy in patient-physician interactions.

### Factors associated with online health information seeking

To the best of our knowledge, no previous study has examined factors associated with online health information seeking among older adults with AF. Our study showed that participants ≥75 years old were less likely to seek health information online compared with those aged 65–74 years, a finding that is consistent with the literature.^24^ In fact, this can be largely due to evidence suggesting that older patients have less confidence in searching for online health information.^24^ In addition, although Internet use has increased dramatically among US adults older than 65 years over the past decade, the use of the Internet is more common among younger adults.^6^ We also found that participants having at least a college degree were more likely to see online health information vs adults with less education, which is consistent with previous studies of middle-aged and older adults with heart disease.^22^^,^^25^ Furthermore, in the present study, participants on anticoagulation therapy were less likely to engage in online health-seeking activities as compared with participants not on OAC. We postulate that participants on OAC may have had prior access to information regarding AF, complications of AF, and OAC use for stroke prevention, specifically during initiation of OAC treatment, which mainly involves a patient-physician shared decision-making process.^26^

### Online health information seeking and AF-related QOL

We found that older adults with AF who sought health information online during the past 4 weeks were more likely to report low AF-related QOL compared with online patients who did not look to the Internet for health information. Given the cross-sectional nature of the current analysis, we cannot tease out whether information seeking improves AF-related QOL or whether those with lower AF-related QOL turn to the Internet for information and advice on how to manage their AF.

One of the main benefits of online health information seeking includes improvements in health outcomes.^27^, ^28^, ^29^ In a cross-sectional study of 1841 adult patients with cancer, those who used Internet health resources reported better health status than those who did not.^27^ Differences between our findings and the results of previous studies could be related to differences in study sample size, sociodemographic and clinical characteristics, and the approach used to assess online health information seeking. Clinicians should provide patients with AF who seek health information online a list of online resources and enhanced educational handouts that have been shown to improve patient understanding and reduce unnecessary health-seeking behaviors, thereby improving the QOL of patients with AF.^30^ In addition, health care providers should encourage patients with AF who seek health information online to engage in online social support systems that have been associated with improved health outcomes.^29^

### Online health information seeking and self-reported efficacy in patient-physician interactions

We found that older adults with AF who sought health information online in the past 4 weeks were less likely to report higher perceived efficacy in their physician interactions than older adults who did not report recent online health information seeking. This finding may reflect the possibility that patients who bring information found online to their clinic appointments have their physicians dismiss their information-seeking efforts and feel less confident to engage with their health care team going forward. Alternatively, it may be that patients with high perceived efficacy for patient-provider interactions advocate for themselves during medical visits, without the need to go online for their health-related information, and have fewer unanswered questions about their health than patients with lower perceived efficacy for patient-provider interactions. Future longitudinal research could examine whether low or reduced efficacy for patient-provider interactions prompts patients to look online to get information to answer their unanswered questions, or whether online health information seeking leads to lower perceived efficacy for communication, perhaps mediated by the provider’s response to the fruits of online searches. Future qualitative studies may also be able to provide insights on the interplay between online health information seeking and dynamics of patient-provider communication.

Multiple factors can affect the patient-physician interaction. If patients’ online findings do not align with physicians’ diagnoses or treatment, this can lead to decreased satisfaction in their physician.^7^^,^^31^^,^^32^ The strengthening or weakening of patients’ relationships with their health care providers depend on their current established relationship as well as whether they have previously discussed their health information.^33^ In addition, with established trust, patients can consult their health care providers for the accuracy of patient-found online health information, which can further strengthen the patient-doctor relationship.^34^ As patients’ online health information seeking becomes extensive and pervasive, the needs and expectations of patients who seek health information online in their interactions with their providers are expected to change.

Health care providers are encouraged to investigate the information patients receive about their health and steer them toward evidence-based resources.^33^^,^^35^ In addition, clinicians should revisit the traditional models of patient-physician communication strategies and develop tailored interventions to improve patient-physician interaction and enhance the shared decision-making process. Moreover, additional research is needed to understand the barriers and concerns of participants who reported low confidence in physician interactions. It is equally important for caregivers and other health care providers to be included in this process in conjunction with patients with AF.

### Study strengths and limitations

Our study has strengths and limitations that need to be considered in the interpretation of the present results. Data were used from a multicenter, geographically diverse cohort of older adults with AF with detailed sociodemographic, geriatric, and clinical characteristics, who are often excluded from studies of mobile technology and the media. This study is unique in terms of examining AF patients’ QOL and perceived efficacy in their physician interactions according to whether they sought health information online. Furthermore, we used standardized, validated instruments to examine AF-related QOL (AFEQT) and self-reported efficacy of patient-physician interaction (PEPPI), increasing the reproducibility of our findings. However, it is important to note that our sample had limited ethnic diversity, with approximately 91% being non-Hispanic White, limiting the generalizability of our findings to older adults of other races/ethnicities. In addition, detailed questions about what type of health information participants sought online were not included in the baseline interview. Hence, we cannot conclude whether participants were seeking information related to their AF and its treatment. In the current study, we only asked online patients (ie, those who had used the Internet in the past 4 weeks) about online health information seeking and did not ask online or offline patients about whether their spouse or other family member had looked online for health information on their behalf (ie, surrogate information seeking).^36^^,^^37^ In the clinical setting, care teams should query not just about digital health activities the patient engages in, but about whether the patient’s spouse or other family members provide “technical support” for their health information needs. Finally, we cannot make causal inferences or determine the directionality of these associations, owing to the cross-sectional nature of this analysis.

## Conclusion

Older adults with AF who sought health information online were more likely to report low AF-related QOL and less likely to have high efficacy in patient-physician interaction than online patients who did not seek health information online in the past 4 weeks. Health care providers should consider barriers to patient-physician interaction among patients who seek health information online, enhance and encourage shared decision-making, recommend further follow-up, and provide their patients with AF disease education plans and a list of online resources for AF.
